# The Effect of Mild Traumatic Brain Injury on Cerebral Microbleeds in Aging

**DOI:** 10.3389/fnagi.2021.717391

**Published:** 2021-09-30

**Authors:** Luca Toth, Andras Czigler, Peter Horvath, Nikolett Szarka, Balint Kornyei, Arnold Toth, Attila Schwarcz, Zoltan Ungvari, Andras Buki, Peter Toth

**Affiliations:** ^1^Department of Neurosurgery, University of Pecs, Medical School, Pecs, Hungary; ^2^Institute for Translational Medicine, University of Pecs, Medical School, Pecs, Hungary; ^3^Department of Radiology, University of Pecs, Medical School, Pecs, Hungary; ^4^Department of Biochemistry, Reynolds Oklahoma Center on Aging, University of Oklahoma Health Sciences Center, Oklahoma City, OK, United States; ^5^Department of Public Health, Faculty of Medicine, Semmelweis University, Budapest, Hungary; ^6^ELKH-PTE Clinical Neuroscience MR Research Group, Pecs, Hungary

**Keywords:** microhemorrages, aging, cognitive decline, traumatic brain injury (TBI), microvascular injury

## Abstract

A traumatic brain injury (TBI) induces the formation of cerebral microbleeds (CMBs), which are associated with cognitive impairments, psychiatric disorders, and gait dysfunctions in patients. Elderly people frequently suffer TBIs, especially mild brain trauma (mTBI). Interestingly, aging is also an independent risk factor for the development of CMBs. However, how TBI and aging may interact to promote the development of CMBs is not well established. In order to test the hypothesis that an mTBI exacerbates the development of CMBs in the elderly, we compared the number and cerebral distribution of CMBs and assessed them by analysing susceptibility weighted (SW) MRI in young (25 ± 10 years old, *n* = 18) and elder (72 ± 7 years old, *n* = 17) patients after an mTBI and in age-matched healthy subjects (young: 25 ± 6 years old, *n* = 20; aged: 68 ± 5 years old, *n* = 23). We found significantly more CMBs in elder patients after an mTBI compared with young patients; however, we did not observe a significant difference in the number of cerebral microhemorrhages between aged and aged patients with mTBI. The majority of CMBs were found supratentorially (lobar and basal ganglion). The lobar distribution of supratentorial CMBs showed that aging enhances the formation of parietal and occipital CMBs after mTBIs. This suggests that aging and mTBIs do not synergize in the induction of the development of CMBs, and that the different distribution of mTBI-induced CMBs in aged patients may lead to specific age-related clinical characteristics of mTBIs.

## Introduction

A traumatic brain injury (TBI) has been shown to induce the formation of cerebral microbleeds (CMBs) (Huang et al., 2015; Toth et al., 2016, 2020; Ungvari et al., 2017; Irimia et al., 2018; Griffin et al., 2019). Cerebral microbleeds are hemosiderin deposits that are 5–10 mm in diameter as a result of bleeding from injured small cerebral arteries, arterioles, or capillaries. They are also associated with the development of cognitive impairments, psychiatric disorders, and gait dysfunctions (Werring et al., 2010; de Laat et al., 2011; Wang et al., 2014, 2018; Huang et al., 2015; Toth et al., 2015, 2020; Akoudad et al., 2016; Ungvari et al., 2017, 2018; Irimia et al., 2018; Nyúl-Tóth et al., 2020). Due to orthostatic hypotension, dehydration, and impaired balance, the elderly population frequently suffers TBIs (Krishnamoorthy et al., 2015; Irimia et al., 2018; Rauen et al., 2020). The most common form of TBI affecting elderly people is mild brain trauma (mTBI) (Rachmany et al., 2013; Krishnamoorthy et al., 2015; Rauen et al., 2020). Similar to TBI, aging is also an independent risk factor for the development of CMBs (Toth et al., 2015, 2020; Ungvari et al., 2017; Irimia et al., 2018). The number of CMBs increases with age, and they are causally linked to age-related cognitive decline and gait disturbances. Interestingly, mechanisms leading to the formation of CMBs, such as cerebrovascular oxidative stress, the activation of matrix metalloproteinases, and the modification of the content of the cerebrovascular wall, are all induced by both aging and TBIs (Lewén et al., 2001; Werring et al., 2010; Rachmany et al., 2013; Abdul-Muneer et al., 2016; Ungvari et al., 2017, 2018; Griffin et al., 2019; Go et al., 2020). However, it is not well established and characterized how TBIs and aging interact to promote the development of CMBs, especially after an mTBI. In this brief study, we tested the hypothesis that an mTBI exacerbates the development of CMBs in the elderly compared with young patients, and aimed to characterize the location and distribution of CMBs in elderly patients after an mTBI.

## Materials and Methods

### Study Population

The study was approved by the Regional Ethic Committee of the University of Pecs, Medical School, Hungary (7270-PTE 2018). We retrospectively analyzed the medical history and three Tesla susceptibility weighted (SWI) MRIs of 35 patients (15 males and 20 females) who had suffered mTBIs [Glasgow Coma Scale (GCS) 14–15] and were admitted to the Department of Neurosurgery, Medical School, University of Pecs, Hungary between April of 2014 and September of 2019. We also analyzed the SWI MRI images of 43 aged-matched control patients (17 males and 26 females) without a medical history of a TBI. For the TBI groups, the inclusion criteria were: young, age is between 18 and 40 years; aged, above 60 years old at the time of the injury; an mTBI in the history within 6 months of the MRI; an mTBI according to Mayo criteria: GCS 14–15, absence or a maximum of 30 min of loss of consciousness, and the absence of post-traumatic amnesia (PTA) (Malec et al., 2007). Exclusion criteria: any conditions associated with CMB formation in the medical history, such as epilepsy, a previous TBI, stroke, transient ischemic attack, cavernous malformations, cerebral amyloid angiopathy, chronic hypertensive encephalopathy, acute haemorrhagic leukoencephalitis, cerebral autosomal dominant arteriopathy with subcortical infarcts and leukoencephalopathy (CADASIL), Alzheimer’s disease, cerebral vasculitis, cerebral metastases, haemorrhagic micrometastases, intracranial embolism, intravascular lymphoma, posterior reversible encephalopathy syndrome (PRES), progressive facial haemiatrophy, thrombotic microangiopathies, intracranial infection, and COL4A1 brain small-vessel disease (Greenberg et al., 2009; Yakushiji, 2015; Ungvari et al., 2017). For the control group, an additional exclusion criterion was a TBI in the medical records. Both in the TBI and control groups, two age groups were defined in a 2 × 2 study design: young (Y): *n* = 20, 10 females, 10 males, age: 25 ± 6 years; young + mTBI (Y + mTBI): *n* = 17, 11 females, 6 males, age: 25 ± 10 years; aged (A): *n* = 23, 16 females, 7 males, age: 68 ± 5 years; aged + mTBI (A + mTBI): *n* = 17, 9 females, 8 males, age: 72 ± 7 years.

### Imaging Protocol

A brain MRI was performed using 3T (Magnetom Trio/Prismafit) Siemens MR scanners (Siemens, Munich, Germany). Susceptibility- and T1-weighted magnetization-prepared rapid acquisition with gradient echo (MPRAGE) and fluid-attenuated inversion recovery (FLAIR) images were obtained. The T1-weighted high-resolution images were then obtained using a three-dimensional (3D) MP-RAGE sequence [TI = 900–1,100 ms; TR = 1,900–2,530 ms; TE = 2.5–2.4 ms; slice thickness = 0.9–1 mm; field of view (FOV) = 256 mm × 256 mm; matrix size = 256 × 256], while 3D SWI images were acquired as follows: TR = 27–49 ms; TE = 20–40 ms; slice thickness = 1.2–3 mm; FOV = 137–201 mm × 230–240 mm; matrix size = 125–182 × 256–320, with no inter-slice gap. For image evaluation, the 3D Slicer 4.8.1^1^ software was used.

### Microbleed Analysis

**FIGURE 1:**
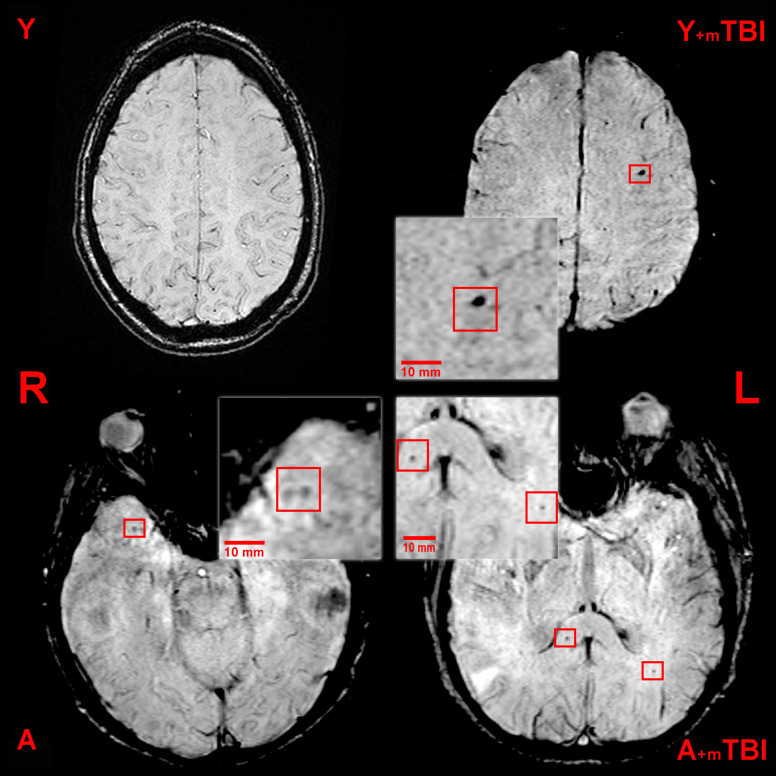
Axial susceptibility weighted (SWI) MRI (three Tesla) of a young control patient (Y, 38-year-old, male), a young patient following mild traumatic brain injury (Y + mTBI, 36-year-old male, GCS: 15), an aged control patient (A, 67-year-old male), and an aged patient with a mild TBI (A + mTBI, 65-year-old male, GCS: 15). Cerebral microbleeds (CMBs) appear as ovoid, hypointense lesions and are indicated by the red squares (R, right; L, left).

Three independent neuroradiologists evaluated the images individually, blinded to medical history. In order to precisely identify CMBs, the exclusion of SWI lesions that mimic CMBs (intersection of veins, bottom of sulci, calcium deposits, artifacts caused by air-tissue interfaces, or macroscopic bleeding caused by, e.g., an intraventricular drain) was carried out (Greenberg et al., 2009; Yakushiji, 2015). The number and location of CMBs were obtained according to the clinically validated Microbleed Anatomic Rating Scale (MARS) (Gregoire et al., 2009). This scale distinguishes the number of definite and possible lesions and precisely localizes the CMBs according to anatomic regions as follows: (1) infratentorial: brainstem or cerebellum; (2) deep: basal ganglia, thalamus, internal or external capsule, corpus callosum, or either the periventricular or deep white matter; (3) lobar: cortex or subcortical white matter. In this study, we present only the definite lesions (Figure 1).

### Statistical Analysis

A Kolmogorov–Smirnov test was performed to determine whether the sample data have the characteristics of a normal distribution. In order to compare the presence of microbleeds, the number of lesions, and specific distribution in different sample groups, Kruskal–Wallis with *post hoc* Dunn’s multiple comparison tests and Mann–Whitney *U* tests were performed. To evaluate the effect of comorbidities on the number of CMBs, Fisher’s exact tests were performed. Differences were considered significant at *p* < 0.05. Statistical analysis was performed using the Origin Pro 2018 software.

## Results

### The Effect of Mild Traumatic Brain Injury on the Formation of Cerebral Microbleeds in Aging

The characteristics of the patients in each group are shown in Table 1. There were no differences in the assessed cerebrovascular risk factors between the groups.

**TABLE 1:** General description and main cardiovascular comorbidities of the study groups.

Group	Age (Mean ± SD)	Sex		Hypertension		Smoking		Urea		Creatinine		Total cholesterol		Low density lipoprotein	
								
		Female	Male	Yes	No	Yes	No	Normal	Abnormal	Normal	Abnormal	Normal	Abnormal	Normal	Abnormal
Young control (Y)	25.09 ± 5.63	50%	50%	10.0%	90.0%	5.0%	95.0%	85.0%	15.0%	85.0%	15.0%	90.0%	10.0%	95.0%	5.0%
Young trauma (Y + mTBI)	24.65 ± 10.22	61.1%	35.3%	5.88%	94.12%	0%	100%	88.24%	11.76%	76.47%	26.53%	94.12%	5.88%	100.0%	0%
Aged control (A)	68.36 ± 4.88	69.6%	30.4%	60.87%	39.13%	4.35%	95.65%	91.3%	8.7%	91.3%	8.7%	56.52%	48.43%	78.26%	21.74%
Aged trauma (A + mTBI)	71.86 ± 7.31	52.9%	47.1%	88.24%	11.76%	17.65%	82.35%	82.35%	17.65%	52.94%	47.06%	82.35%	17.65%	100.0%	0%

**FIGURE 2:**
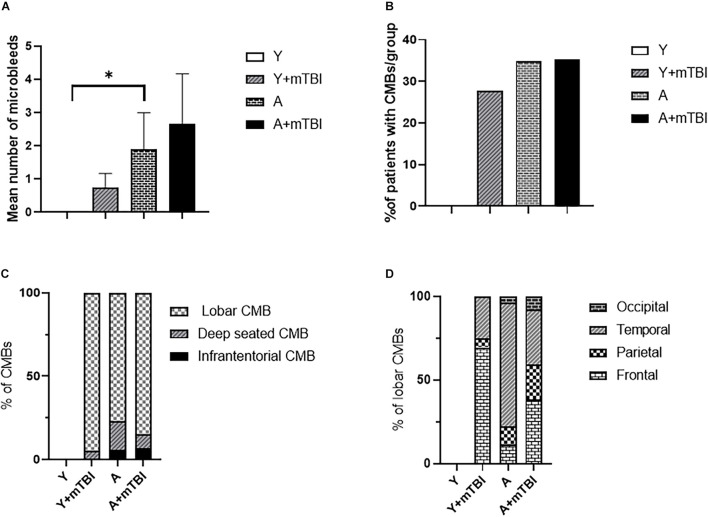
Effect of mild traumatic brain injury on the development and characteristics of cerebral microbleeds in the elderly. **(A)** Mean number of CMBs in young control (Y) patients (*n* = 20, age: 25.09 ± 5.63 years), young patients after an mBTI (Y + mTBI) (*n* = 17, age: 24.65 ± 10.22 years), aged control patients (A) (*n* = 23, age: 68.36 ± 4.88 years), and aged patients with mTBIs (A + mTBI, *n* = 17, age: 71.86 ± 7.31 years). Data are mean ± SEM, ^∗^*P* < 0.05 vs. YC, ns: non-significant. **(B)** Number of patients with CMBs in the studied groups is expressed as the percent of the total number of patients in each group [young control (Y) patients (*n* = 20, age: 25.09 ± 5.63 years), young patients after mBTIs (Y + mTBI) (*n* = 17, age: 24.65 ± 10.22 years), aged control patients (A) (*n* = 23, age: 68.36 ± 4.88), and aged patients with mTBIs (A + mTBI) (*n* = 17, age: 71.86 ± 7.31 years)]. ^∗^*P* < 0.05 vs. YC. Panel **(C)** depicts the localization of CMBs in each group as number of lobar, deep-seated (basal ganglion), and infratentorial CMBs expressed as the percent (%) of the total number of CMBs. Note that the majority of CMBs can be found supratentorially (lobar and basal ganglion); however, a small number of microbleeds appear in the infratentorial location in aged patients after an mTBI. The difference did not reach statistical significance. **(D)** Lobar distribution of supratentorial CMBs in each studied group of patients (frontal, temporal, parietal, and occipital). Please note that aging enhances the number of parietal and occipital CMBs after an mTBI (*P* < 0.05 vs. Y + mTBI), and that an mTBI leads to the formation of more CMBs in the frontal, parietal, and occipital lobes in aging (*P* < 0.05 vs. A). (Y): *n* = 20, 10 females, 10 males, age: 25.09 ± 5.63 years; young + mTBI (Y + mTBI): *n* = 17, 11 females, 6 males, age: 24.65 ± 10.22 years; aged (A): *n* = 23, 16 females, 7 males, age: 68.36 ± 4.88 years; aged + mTBI (A + mTBI): *n* = 17, 9 females, 8 males, age: 71.86 ± 7.31 years.

We found that aging exacerbated the formation of CMBs significantly (*p* < 0.05) compared with young patients (Figure 2A), confirming the results of previous studies showing that aging is an independent risk factor for the development of CMBs (Greenberg et al., 2009; Toth et al., 2015; Irimia et al., 2018). Importantly, the number of CMBs in elderly patients was not further increased by mTBIs (Figure 2A). An mTBI did not enhance the number of CMBs in young patients either (Figure 2A). We found that aging also significantly exacerbated (*p* < 0.05) the incidence of patients with CMBs regardless of the number of bleedings (percent of patients with CMBs in the given group of patients) compared with the young patients (Figure 2B), who were not affected by mTBIs (Figure 2B).

### Location Characteristics of Aging and Mild Traumatic Brain Injury-Induced Cerebral Microbleeds

We found the majority of CMBs in the supratentorial compartment (lobar and basal ganglion); however, a small number of microbleeds appear in the infratentorial location in aged patients after an mTBI. The difference did not reach statistical significance (Figure 2C). Analysing the distribution of supratentorial CMBs across cerebral lobes (frontal, temporal, parietal, and occipital), we found that aging enhances the number of parietal and occipital CMBs after an mTBI (*P* < 0.05 vs. Y + mTBI), and that an mTBI leads to the formation of more CMBs in the parietal lobes in aging (*P* < 0.05 vs. A; Figure 2D).

## Discussion

It has been shown previously that both TBIs and aging induce the development of CMBs (Huang et al., 2015; Toth et al., 2015; Ungvari et al., 2017; Irimia et al., 2018; Wang et al., 2018; Griffin et al., 2019). In both cases, CMBs are associated with long-term cognitive deficits and gait dysfunctions and determine the outcome of patients (Werring et al., 2010; de Laat et al., 2011; Huang et al., 2015; Toth et al., 2015, 2020; Yakushiji, 2015; Akoudad et al., 2016; Ungvari et al., 2017, 2018; Irimia et al., 2018; Nyúl-Tóth et al., 2020). Previous epidemiological studies have proposed that the TBI-related development of CMBs is exacerbated in aging (Irimia et al., 2018). However, the effect of an mTBI, which is the most frequent form of brain trauma, on the development of CMBs in aging, has not been established (Wang et al., 2014; Krishnamoorthy et al., 2015; Rauen et al., 2020). Here, we show (Figure 2) that significantly more microbleeds can be found in aging human brains than in the brains of young healthy individuals, confirming the results of previous studies (Greenberg et al., 2009; Werring et al., 2010; Toth et al., 2015; Akoudad et al., 2016; Wang et al., 2018). We also found significantly more CMBs in elderly patients after an mTBI compared with young patients with an mTBI; however, we did not observe a significant difference in the number of cerebral microhemorrhages between aged and aged patients with mTBIs. This suggests that aging and mTBIs do not synergize the induction of the development of CMBs.

The clinical consequences of CMBs, such as the development of cognitive decline, are most likely due to the cumulative effects of the lesions and the damage in specific anatomical locations (Werring et al., 2010; Ding et al., 2017; Ungvari et al., 2017). For example, damage to the fronto-subcortical circuits linking prefrontal areas to basal ganglia is associated with impairments in executive function, and the disarrangement of pathways from the mentioned areas projecting to the thalamus results in memory disturbances (Werring et al., 2010; Ding et al., 2017; Levit et al., 2020). Although morphological characteristics based on MRI examinations are not helpful in distinguishing CMBs of different etiologies, specific locations suggest the pathophysiological reasons of CMB formation (Greenberg et al., 2009; Wang et al., 2014; Yakushiji, 2015; Ding et al., 2017; Ungvari et al., 2017). For example, the typical brain areas for traumatic CMBs are the corona radiata and longitudinal fasciculus (Wang et al., 2014; Ungvari et al., 2017; Toth et al., 2020). Cerebral microbleeds in deep cerebral areas are thought to be due to cerebral angiopathy induced by hypertension, and lobar CMBs are likely due to amyloid angiopathy (Wang et al., 2014; Yakushiji, 2015; Ding et al., 2017; Ungvari et al., 2017). We found that aging alters the distribution of CMBs after an mTBI (Figure 2), namely, in elderly patients following an mTBI, the number of occipital and parietal bleedings was exacerbated compared with young patients. This may affect the functional consequences of these bleedings. Accordingly, the occipital and parietal lobes are responsible for integrating visual and cognitive information, and play an important role in voluntary coordination, posture and motor control, spatial cognition, and the rapid corrections of movements (Galletti et al., 2003; Niogi et al., 2008; Freedman and Ibos, 2018; MacKinnon, 2018). Specific tests should be part of the patient characterization after an mTBI to assess the region-specific consequences of CMBs in aging (and also in young patients), such as the trail making test, Beck’s depression test, and Montreal Cognitive Assessment test. This possibility should be verified in the future.

### Limitations and Perspectives

The major limitations of this study are its retrospective design and relatively small sample size. Future prospective studies should verify the findings of this study with a large number of healthy control volunteers. We used the Mayo criteria to define an mTBI. Since other guidelines suggest slightly different scoring systems, it would be important to compare the CMB formation in TBI groups as defined by various scoring systems. Aging and mTBIs may interact in altering regulatory mechanisms of cerebral blood flow (CBF) in a functional manner. Accordingly, the changes in neurovascular coupling, autoregulation of CBF, and cerebrovascular reactivity should be assessed and correlated with the cognitive and gait functions in different age groups after mTBIs. Finally, the possible mechanisms through which aging and TBIs may interact to alter cerebrovascular function and the formation of CMBs should be studied, with a special focus on mitochondrial oxidative stress, the activation of redox-sensitive matrix metalloproteinases, the modification of the cerebrovascular wall, the production of proinflammatory cytokines, and the disruption of the blood-brain barrier (Lewén et al., 2001; Greenberg et al., 2009; Werring et al., 2010; de Laat et al., 2011; Toth et al., 2015, 2020; Yakushiji, 2015; Ungvari et al., 2018; Levit et al., 2020).

## Data Availability Statement

The original contributions presented in the study are included in the article/supplementary material, further inquiries can be directed to the corresponding author.

## Ethics Statement

The studies involving human participants were reviewed and approved by the study was approved by the Regional Ethic Committee of the University of Pecs, Medical School, Hungary (7270-PTE 2018). Written informed consent for participation was not required for this study in accordance with the national legislation and the institutional requirements.

## Author Contributions

LT and PT designed the studies and protocols. AC, PH, BK, and NS performed the literature search and collected the patient data. LT, AC, BK, and AT performed the image analysis. LT, AC, and NS generated the figures. LT, AC, and PT wrote the manuscript. LT, AC, PH, BK, NS, AT, AS, ZU, AB, and PT edited and revised the manuscript. All authors contributed to the article and approved the submitted version.

## Conflict of Interest

The authors declare that the research was conducted in the absence of any commercial or financial relationships that could be construed as a potential conflict of interest.

## Publisher’s Note

All claims expressed in this article are solely those of the authors and do not necessarily represent those of their affiliated organizations, or those of the publisher, the editors and the reviewers. Any product that may be evaluated in this article, or claim that may be made by its manufacturer, is not guaranteed or endorsed by the publisher.

## Footnotes

1
http://www.slicer.org
